# Multimodal Performance of GPT-4 in Complex Ophthalmology Cases

**DOI:** 10.3390/jpm15040160

**Published:** 2025-04-21

**Authors:** David Mikhail, Daniel Milad, Fares Antaki, Jason Milad, Andrew Farah, Thomas Khairy, Jonathan El-Khoury, Kenan Bachour, Andrei-Alexandru Szigiato, Taylor Nayman, Guillaume A. Mullie, Renaud Duval

**Affiliations:** 1 Temerty Faculty of Medicine, University of Toronto, Toronto, ON M5S 1A1, Canada; d.mikhail@mail.utoronto.ca; 2 Department of Ophthalmology, University of Montreal, Montreal, QC H3T 1J4, Canadajonathan.el-khoury@umontreal.ca (J.E.-K.); kenan.bachour@umontreal.ca (K.B.);; 3 Department of Ophthalmology, Hôpital Maisonneuve-Rosemont, Montreal, QC H1T 2M4, Canada; 4 Cole Eye Institute, Cleveland Clinic, Cleveland, OH 44195, USA; 5 CHUM School of Artificial Intelligence in Healthcare (SAIH), Centre Hospitalier de l’Université de Montréal (CHUM), Montreal, QC H2X 3E4, Canada; 6 Department of Software Engineering, University of Waterloo, Waterloo, ON N2L 3G1, Canada; jmilad@uwaterloo.ca; 7 Faculty of Medicine, McGill University, Montreal, QC H3A 0G4, Canada; 8 Department of Ophthalmology, Hôpital du Sacré-Coeur de Montréal, Montreal, QC H4J 1C5, Canada; 9 Department of Ophthalmology, St. Mary’s Hospital Center, Montreal, QC H3T 1M5, Canada

**Keywords:** artificial intelligence, LLM, multimodal, ophthalmology

## Abstract

**Objectives:** The integration of multimodal capabilities into GPT-4 represents a transformative leap for artificial intelligence in ophthalmology, yet its utility in scenarios requiring advanced reasoning remains underexplored. This study evaluates GPT-4’s multimodal performance on open-ended diagnostic and next-step reasoning tasks in complex ophthalmology cases, comparing it against human expertise. **Methods**: GPT-4 was assessed across three study arms: (1) text-based case details with figure descriptions, (2) cases with text and accompanying ophthalmic figures, and (3) cases with figures only (no figure descriptions). We compared GPT-4’s diagnostic and next-step accuracy across arms and benchmarked its performance against three board-certified ophthalmologists. **Results**: GPT-4 achieved 38.4% (95% CI [33.9%, 43.1%]) diagnostic accuracy and 57.8% (95% CI [52.8%, 62.2%]) next-step accuracy when prompted with figures without descriptions. Diagnostic accuracy declined significantly compared to text-only prompts (*p* = 0.007), though the next-step performance was similar (*p* = 0.140). Adding figure descriptions restored diagnostic accuracy (49.3%) to near parity with text-only prompts (*p* = 0.684). Using figures without descriptions, GPT-4’s diagnostic accuracy was comparable to two ophthalmologists (*p* = 0.30, *p* = 0.41) but fell short of the highest-performing ophthalmologist (*p* = 0.0004). For next-step accuracy, GPT-4 was similar to one ophthalmologist (*p* = 0.22) but underperformed relative to the other two (*p* = 0.0015, *p* = 0.0017). **Conclusions**: GPT-4’s diagnostic performance diminishes when relying solely on ophthalmic images without textual context, highlighting limitations in its current multimodal capabilities. Despite this, GPT-4 demonstrated comparable performance to at least one ophthalmologist on both diagnostic and next-step reasoning tasks, emphasizing its potential as an assistive tool. Future research should refine multimodal prompts and explore iterative or sequential prompting strategies to optimize AI-driven interpretation of complex ophthalmic datasets.

## 1. Introduction

Artificial intelligence (AI) in ophthalmology is gaining research interest, particularly for clinical integration foundation models. Large AI systems based on deep neural networks and self-supervised learning can be adapted to various tasks with minimal retraining [1,2]. Large language models (LLMs), such as Generative Pre-trained Transformer (GPT), are a type of foundation model trained on textual data and excel in natural language processing [3]. Advances in LLMs like Generative Pre-trained Transformer (GPT) offer advanced natural language processing, using textual input to create human-like responses. Previous studies on GPT in ophthalmology have focused on text-based input and multiple-choice tasks [4,5]. Despite this growing body of work, much of the research to date has concentrated on text-only interactions or performance on multiple-choice questions. Several studies have benchmarked GPT-4 and its predecessors against board examination questions or standardized knowledge assessments. For instance, ChatGPT-4 has shown variable performance in medical education tasks—outperforming legacy versions and sometimes matching or exceeding human examinees on simpler, text-based prompts. However, its performance tends to decline in tasks requiring interpretation of images, complex reasoning, or synthesis of multimodal information [6].

We previously assessed GPT-4, a state-of-the-art LLM, using a dataset from the JAMA Ophthalmology Clinical Challenges, which includes 422 real-world, complex cases [7]. Readers are tasked with determining the most likely diagnosis (open-ended) and the correct next step (multiple-choice). Using text-only prompts, GPT-4 achieved 47.9% accuracy in diagnosis and 63.0% in next-step tasks, comparable to ophthalmologists. Recently, GPT-4’s vision capabilities were introduced, enabling the input of interleaved image–text prompts.

Given the essential role of ophthalmic imaging in diagnosing and managing ocular pathologies, GPT-4’s new multimodal capabilities warrant further exploration. In this study, we assessed GPT’s performance on multimodal ophthalmic cases using the JAMA Ophthalmology Clinical Challenges dataset, which contains both multiple-choice and open-ended diagnostic reasoning problems [8]. We compared quantitative performance metrics on open-ended and multiple-choice questions with our previous text-only prompts study.

The objective of this study was to evaluate GPT-4’s diagnostic and clinical reasoning performance in complex ophthalmology cases when provided with different combinations of textual and image-based information. Specifically, we aimed to assess how the inclusion or exclusion of figure descriptions influences its ability to interpret and reason through real-world clinical challenges and how this performance compares to that of practicing ophthalmologists.

## 2. Materials and Methods

### 2.1. JAMA Ophthalmology’s Clinical Challenges

This was a cross-sectional study adhering to the Strengthening the Reporting of Observational Studies in Epidemiology (STROBE) reporting guideline. We used JAMA Ophthalmology’s Clinical Challenges section, consisting of 422 cases until 3 July 2024. These cases test diagnosis with open-ended questions and next-step predictions with multiple-choice questions. We categorized each case into 1 of 13 ophthalmology subspecialties, defined by the American Academy of Ophthalmology’s Basic and Clinical Science Course [9].

### 2.2. Study Arms

**Figure 1:**
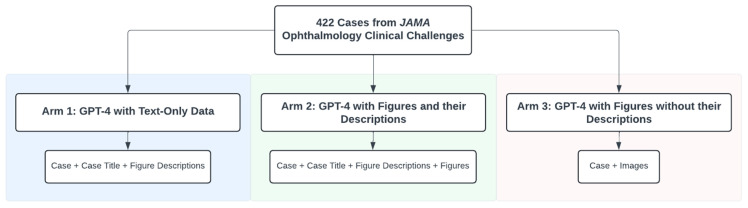
The three study arms and the data used as prompts.

We prompted ChatGPT on three separate occasions, each representing an arm of this study. The first arm was a text-only prompt containing the case, case title, and figure descriptions. In the second arm, we prompted GPT-4 with a text prompt, including the case, case title, and figure descriptions, along with their associated figures between 21 February 2024 and 16 March 2024. In the third arm, the prompt includes solely the case and figures, excluding the legends and any description of the figure findings in the case title. Figures were typically external photographs of pathology or diagnostic photos images such as fundus photographs, slitlamp photographs, or optical coherence tomography (OCT) scans. We performed these prompts between 9 June 2024 and 3 July 2024. Figure 1 summarizes the different arms of this study and the data used as prompts.

### 2.3. GPT-4 Access

We used “gpt-4-turbo” trained on data until December 2023. It supports multimodal input with a 128,000-token limit, allowing full case uploads. We used ChatGPT instead of the application programming interface (API) for easier interaction and follow-up prompts, utilizing GPT-4’s self-correction ability. Since the API was not used, we could not specify the exact temperature, but it is estimated to be around 0.7, balancing creativity and coherence [10,11].

A new ChatGPT Plus account was created to avoid prior chat history influencing responses. Three independent reviewers (D. Mikhail, A.F., T.K.) handled prompts and data extraction. The “chat history and training” setting was disabled, and new sessions were started for each case to reduce memory retention bias. No institutional review board approval was needed as no human subjects were involved.

We compared GPT-4’s responses to the original case answers, prioritizing specificity in grading diagnoses. At least two independent reviewers (D. Mikhail, A.F., T.K., and D. Milad) recorded and reviewed responses, confirming if a definitive diagnosis and correct answers were provided. An answer was correct only if both the general diagnosis and specific etiology were identified. For example, “uveitis” was insufficient if the etiology was “syphilitic uveitis”.

### 2.4. Prompting Algorithm

The Zero-Shot Plan-and-Solve + (PS+) prompt was found to be most effective in our prior analysis and was chosen for this study. Wang et al. devised the PS+ prompt by having GPT divide its task into, executed sequentially with detailed instructions [12]. There was only one prompt per case. Each case had one prompt, which included the full clinical case, a multiple-choice question, and answer options. The questions followed a standard format with one correct answer and three distractors.

**Figure 2:**
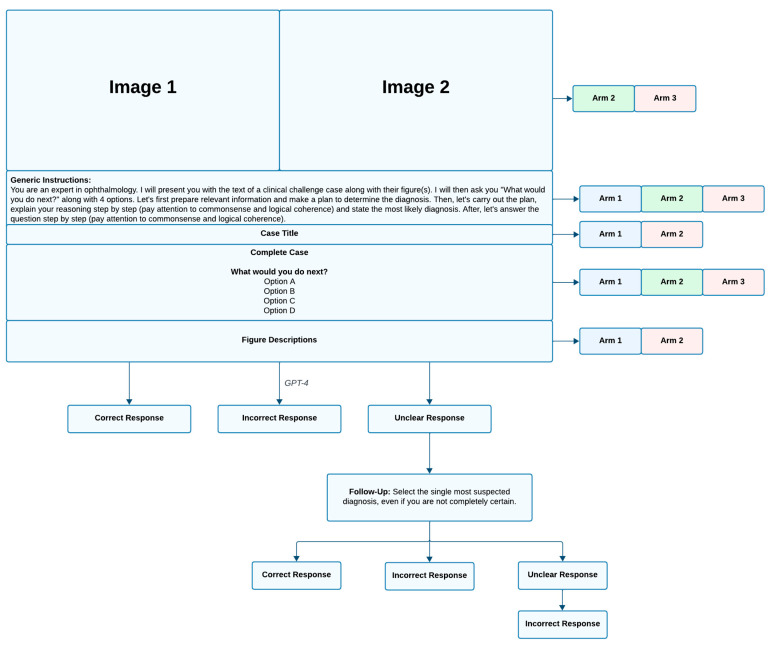
Prompting algorithm containing sample prompt and response of GPT-4.

If GPT-4’s initial response lacked a definitive diagnosis, it was labeled “unclear” and prompted for self-correction with: “Select the single most suspected diagnosis, even if uncertain”. This encouraged GPT-4 to provide specific diagnoses instead of tentative ones. Cases requiring self-correction were recorded, and if no definitive diagnosis was given, they were marked incorrect. Correct diagnoses after follow-up were considered correct, with self-correction noted. The prompting algorithm and a sample prompt are shown in Figure 2.

### 2.5. Human Benchmarking

Three board-certified ophthalmologists specializing in comprehensive ophthalmology, glaucoma, and medical retina served as human comparators. To reduce bias, only participants who had not contributed to case preparation and had no prior exposure to the selected cases were included. Each answered five randomly selected cases from 10 ophthalmology subspecialties, totaling 47 cases (including 2 General Medicine cases). They received identical prompts with cases and figures, were blinded to the correct answers, and their responses were recorded in an Excel spreadsheet.

### 2.6. Statistical Analysis

The primary outcome was the diagnostic accuracy of GPT-4 and its accuracy on the next-step task, calculated as the weighted mean proportion of correct answers from all subspecialties. The accuracy and 95% confidence interval (CI) were calculated using the Wilson Score Interval (Table S1).

We hypothesize that GPT-4 will demonstrate high diagnostic accuracy and effectiveness in determining the next-step task, comparable to ophthalmologists’ performance. We also posit that incorporating ophthalmic figures into the prompts will enhance diagnostic performance.

Secondary endpoints included comparing GPT-4’s performance with and without figure descriptions to our prior text-only analysis and to the performance of three ophthalmologists. We used chi-squared (χ^2^) tests to compare proportions. Confusion matrices were employed to assess GPT-4’s performance on diagnosis and next-step tasks (Tables S3–S5). We also analyzed how subspecialty, number of images per figure, and imaging modality affected the correctness of each arm.

Fleiss’ kappa (κ) was utilized to quantify the agreement between human graders. Kappa values can be classified as: 0–0.20 (none to slight agreement), 0.21–0.40 (fair agreement), 0.41–0.60 (moderate agreement), 0.61–0.80 (substantial agreement), and 0.81–1.00 (near perfect agreement). Human graders were tested on a subset of the clinical challenges. Of the total number of cases tested on GPT-4, only this subset of cases was used to recalculate the LLM’s performance to ensure a fair comparison with human graders; thus, the performance values may differ compared to those in the previous section.

## 3. Results

The most common subspecialties of the 422 JAMA Clinical Challenges dataset included Retina and Vitreous, Uveitis, and Neuro-Ophthalmology, comprising 96 (23%), 67 (16%), and 67 (16%) cases, respectively. The remaining subspecialties and their proportions are in Figure S1. No challenges were published on Refractive Surgery, Clinical Optics, and Fundamentals.

### 3.1. GPT-4 Without Figure Descriptions Requires Less Self-Corrective Prompting

On average, each case had 2.05 figures used as prompts alongside the clinical case. There were 218 cases (51.7%) requiring self-correction with figure descriptions, compared to 174 cases (41.2%) without descriptions (*p* = 0.002). Table S1 summarizes GPT-4’s follow-up prompting accuracy by subspecialty and figure use. A two-proportion z-test showed that Pathology and Tumors cases without figure descriptions (*p* = 0.017) and Glaucoma cases with descriptions (*p* = 0.014) were more likely to need follow-up. There were no significant differences in correct diagnoses after follow-up, and next-step responses remained unchanged.

### 3.2. GPT-4 Underperforms with Ophthalmic Figures Versus Text-Only Prompts

GPT-4 using ophthalmic figures without descriptions achieved a diagnostic accuracy of 38.4% (95% CI [33.9%, 43.1%]) and a next-step accuracy of 57.8% (95% CI [52.8%, 62.2%]). In comparison, GPT-4 using text-only prompts reached 47.9% (95% CI [43.1%, 52.9%]) for diagnosis and 63.0% (95% CI [58.2%, 67.6%]) for next-step accuracy. Incorporating figures significantly reduced diagnostic accuracy (*p* = 0.007) but did not affect next-step accuracy (*p* = 0.140) (Tables S3–S5). Subspecialty analysis showed worse performance with figures in diagnosing Pathology and Tumors (*p* = 0.0049) and Pediatric Ophthalmology (*p* = 0.013) compared to text-only prompts (Table 1).

### 3.3. Adding Figure Descriptions Enhanced Diagnostic Accuracy of Image-Based Prompts

**Figure 3:**
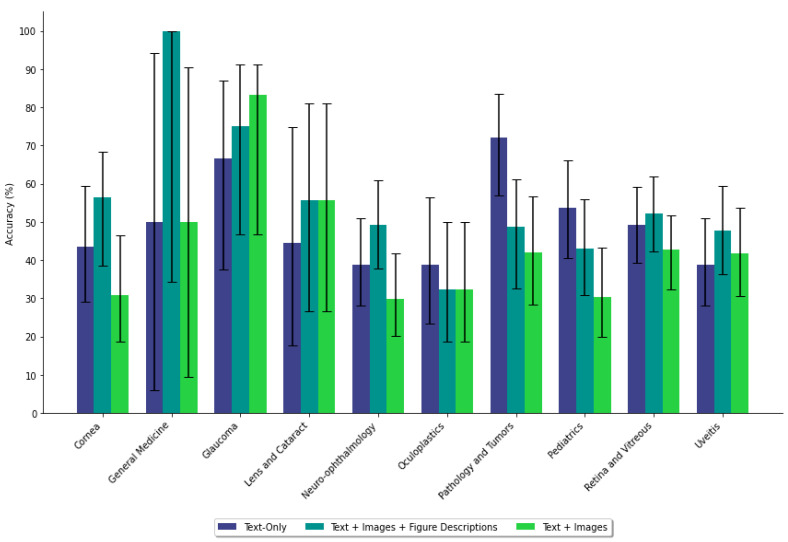
Diagnostic accuracy of GPT-4 across ophthalmology subspecialties under three prompting conditions: text-only, text with figures and descriptions, and text with figures alone. Each subspecialty shows variation in performance across conditions, with a general trend of reduced accuracy when figure descriptions are omitted.

**Figure 4:**
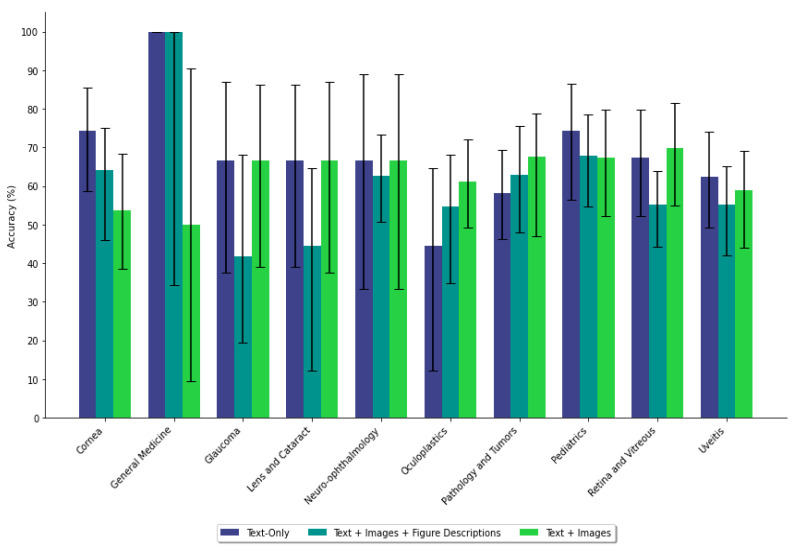
Next-step accuracy of GPT-4 across subspecialties under different prompting formats. Accuracy is generally more consistent than in the diagnosis task, with fewer pronounced differences between arms. Some subspecialties exhibit minor variability, but overall performance remains relatively stable regardless of the presence of the figure description.

When figure descriptions were added, GPT-4 achieved a diagnostic accuracy of 49.3% (95% CI [48.54%, 50.08%]) and next-step accuracy of 59.2% (95% CI [58.61%, 59.88%]). These results were not significantly different from text-only prompts for diagnosis (*p* = 0.684) or next step (*p* = 0.258) (Table 2). Adding descriptions improved diagnostic accuracy compared to figure-only prompts (*p* = 0.0014) but did not affect next-step accuracy (*p* = 0.680). Diagnostic performance improved significantly in Cornea (*p* = 0.024) and Neuro-ophthalmology (*p* = 0.022) cases (Table S2, Figure 3 and Figure 4).

### 3.4. Impact of Figure Images on Accuracy Based on Case Characteristics

We investigated how including figures affected answer correctness based on subspecialty, number of images, and imaging modalities used (Figures S2–S5). Answer correctness was categorized as follows: remained correct, became correct, or became incorrect after adding figures. Retina and Vitreous cases were the most common in all categories. For cases with one image, the most common modalities were external, fundus, and slit lamp photographs. For cases with two images, they typically included either two images of the same type (e.g., external or fundus photographs) or a combination of a fundus photograph and an OCT scan.

### 3.5. GPT-4 Prompted with Images Performs Similarly to Two of Three Ophthalmologists on Diagnosis

**Figure 5:**
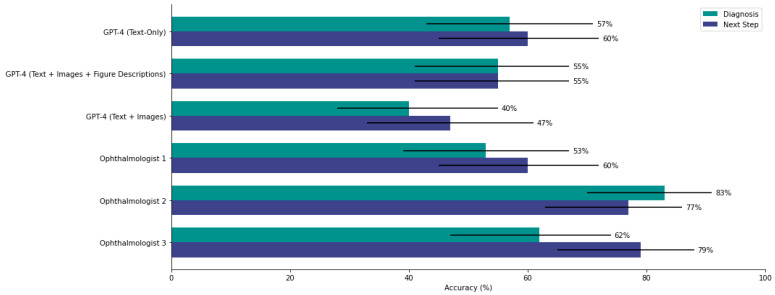
Accuracy comparison between GPT-4 and three board-certified ophthalmologists on a subset of 47 cases. Diagnostic and next-step performances for each participant are displayed. GPT-4’s performance aligns more closely with the lower-performing ophthalmologist, particularly for next-step questions, but trails behind the top-performing clinician in both tasks.

GPT-4 was compared to three board-certified ophthalmologists across 47 cases. Interrater agreement was moderate for diagnosis (κ = 0.43, 95% CI [0.32, 0.55]) and next step (κ = 0.51, 95% CI [0.38, 0.63]), so performance was compared individually. GPT-4, when using images, had diagnostic and next-step accuracies of 40.4% and 46.8%, respectively. The ophthalmologists achieved diagnostic accuracies of 76.6% (*p* = 0.0004), 51.1% (*p* = 0.30), and 48.9% (*p* = 0.41). For next-step accuracy, the ophthalmologists scored 78.7% (*p* = 0.0015), 78.3% (*p* = 0.0017), and 59.6% (*p* = 0.22). GPT-4 performed similarly to the lowest-scoring ophthalmologist in both tasks but significantly worse than the highest scorers (Figure 5).

## 4. Discussion

When prompted with complex ophthalmology cases and images, GPT-4 achieved accuracies of 38.4% for diagnosis (open-ended) and 57.8% for the next step (multiple-choice). The lower diagnostic accuracy may reflect the difficulty of synthesizing case details and images compared to the straightforward, deductive task of answering multiple-choice questions. In comparison, GPT-4 performed better using text-only data, with a higher diagnosis accuracy (47.9%, *p* = 0.007) but similar results for next-step tasks (63.0%, *p* = 0.140). This change in diagnostic accuracy is statistically significant and may be clinically relevant in settings such as emergency triage or remote consultation, where AI-generated differentials could influence early decision-making. Such use cases suggest that LLMs like GPT-4 may offer supportive roles in environments with limited access to subspecialists, including rural or telemedicine contexts. While not yet suitable for autonomous interpretation, their ability to approximate clinical reasoning, particularly with textual prompts, highlights their potential as assistive tools rather than diagnostic replacements.

GPT-4 performed worse in Pathology and Tumor (*p* = 0.0049) and Pediatrics (*p* = 0.013) cases when using figures. One potential reason for this could be limited exposure to biopsy micrograph images and pediatric cases during training. When the case, figures, and figure descriptions were provided, its performance was similar to text-only results for diagnosis (49.3%, *p* = 0.684) and next step (59.2%, *p* = 0.258). Although these differences were not statistically significant, the observed trend may still warrant clinical attention in high-stakes scenarios where even modest improvements in diagnostic precision can impact patient outcomes.

These findings suggest that replacing textual descriptions with figures led to poorer diagnostic performance, revealing GPT-4’s limitations in interpreting ophthalmic figures independent of textual data. OpenAI acknowledges that GPT-4 primarily processes textual data and may lack advanced features for handling imaging data [13]. The similar performance of GPT-4 using text-only versus figures with descriptions suggests that imaging data were likely not utilized to perform the tasks. Some figures included multiple images partitioned by a white bar in the middle, making it difficult for GPT-4 to distinguish between closely spaced images. Further, contextual mixing between images may lead the model to confuse features from one image with another, resulting in misinterpretation and misdiagnosis. Although labels were often embedded within the images themselves, GPT-4 may not have reliably interpreted these visual cues. In cases where figures included both eyes or multiple imaging modalities (e.g., a fundus photo alongside an OCT scan), the model could have perceived them as a single composite image. This could have affected its ability to distinguish laterality, identify the correct sequence, or extract clinically relevant differences between images—factors that may have influenced diagnostic accuracy. This may explain the increased incidence of unclear responses and subsequent self-correction required.

Prior research suggests that GPT-4 performs worse on multiple-choice questions with ophthalmic figures, achieving 65% accuracy. Another study found that GPT-4 was more likely to correctly diagnose multimodal retina cases when patients’ age and sex were included, suggesting reliance on text-based clinical data accompanying the figures [14]. Xu et al. reported poor performance on open-ended tasks, with accurate responses in 30.6% of prompts from a dataset of 60 images [15]. Comparable limitations were observed in radiology, where Kufel et al. found that ChatGPT-3.5 underperformed on Poland’s specialty exam, with reduced accuracy on complex, classification-based questions and stronger performance on clinical management tasks [16]. This mirrors our finding that GPT-4 struggled with diagnostic reasoning in multimodal ophthalmology cases, particularly when deprived of descriptive context. GPT-4 also showed limitations in reliably interpreting nuclear medicine images and orthopedic surgery questions requiring higher-order thinking [17,18]. Our results align with previous findings, as our cases involved complex, rare diagnoses with specific management plans. Our study differed from another, which prompted GPT-4 one figure at a time. It remains unclear how well GPT-4 processes multiple figures in a single prompt compared to prompting in a sequential fashion. Collage prompting is a cost-efficient alternative to standard visual prompting proposed by Xu et al., which concatenates multiple images into the same size to form a single visual prompt [19]. This prompting method reduced unnecessary expenses associated with multiple token-expensive prompts without compromising the accuracy of image interpretation [19]. However, challenges may arise when optimizing the arrangements of images differing in size and quality into a single array.

LLMs like GPT are transformer models built on neural networks, generating content by predicting the next likely “token” (i.e., words, letters, or punctuation) [7,20,21]. In this study, GPT-4 processed more than just token-based inputs. Since OpenAI does not publish ChatGPT’s source code, the mechanism for integrating image and text data remains unclear. GPT-4’s lower diagnostic performance may result from misinterpreted figures that could mislead the model when combined with the case’s textual information. Despite these limitations, several practical strategies may help improve LLM performance in ophthalmology. First, structured visual prompting—such as isolating images one at a time, reducing visual clutter, or adding annotations like laterality—may enhance interpretation accuracy. Domain-specific fine-tuning or integration with retrieval-augmented generation (RAG) models trained on ophthalmic data may substantially boost diagnostic performance [22].

When prompts included figures without descriptions, 41.2% of GPT-4’s initial responses lacked a specific diagnosis, instead speaking in generalities. This required follow-up prompts, an issue not encountered in the original study with text-only data. ChatGPT’s source code may have been updated to be more cautious in offering medical information without clear, specific prompting. Given the role of the PS+ prompting algorithm in our previous paper, prompting methods will likely require continuous refinement as LLMs evolve. A recent study found that few-shot learning prompts outperformed zero-shot learning in classification and biomedical reasoning tasks [23]. Liu et al. customized their own “MetalPrompt”, which outperformed few-shot learning, chain-of-thoughts integrated with few-shot learning, and zero-shot learning in materials science classification tasks [24]. This may suggest the importance of prompt flexibility and engineering to enable customization based on the field of study and target tasks [24].

Comparisons between GPT-4 and the three ophthalmologists should be contextualized by the limited participant size. Since two of the three ophthalmologists were subspecialists, their knowledge may have been more concentrated on one subspecialty compared to the third, a comprehensive ophthalmologist. As GPT-4 and other LLMs improve in handling clinical and image data, their potential to match or surpass ophthalmologists in diagnostic accuracy and treatment plan selection remains to be discovered.

This study has certain limitations. Firstly, it is unknown whether GPT-4 was already trained on data from JAMA Ophthalmology’s Clinical Challenges. If so, this could lead to data leakage, where overlap between training and test data inflates model performance by enabling recall rather than genuine reasoning. While these cases were utilized in our initial study, we did not disclose the correct answers in either study. Further, to prevent GPT-4 from using its “memory”, we created new chats for each case. Nonetheless, without transparency into the models’ training data, it remains difficult to determine whether performance reflects true capability or familiarity with the cases. Secondly, differences in figure quality and formatting may have affected performance, especially with suboptimal images. Like earlier models, GPT-4 is not entirely reliable and can experience “hallucinations”, generating plausible but inaccurate information [25,26]. For instance, GPT-4 may reference imaginary tests in its diagnosis. Thus, researchers, ophthalmologists, and patients should be cautious when using GPT-4 for medical advice and avoid inputting sensitive information. Thirdly, the human benchmarking component involved only three ophthalmologists evaluating 47 cases. This limited sample size reduces the statistical power of our comparisons and increases the risk of overinterpreting *p*-values. As such, performance differences between GPT-4 and human participants should be interpreted with caution. Larger validation studies with a larger number of clinician participants are needed to draw more definitive conclusions.

To conclude, GPT-4 performed worse on the diagnosis task when prompted with the full case, including ophthalmic figures, compared to our previous study with text-only prompts. However, performance on the next-step task remained similar. Future work should assess whether GPT-4 performs better with multiple figures in a single prompt or when prompted stepwise. Future applications of GPT-4 in ophthalmology may include triage support, clinical education, and second-opinion tools, but these must be carefully evaluated for reliability, particularly in image-dependent cases. Appropriate applications of the LLM in clinical settings must be thoughtfully considered within the model’s confines as an assistive tool, given the medicolegal and bioethical risks involved.

## Figures and Tables

**Table 1:** Comparing the performance of GPT-4 using figures without figure descriptions and GPT-4 using text-only data on diagnosis and next-step tasks by subspecialty.

Prompting Data	Subspeciality	Diagnosis			Next Step		
		Mean (%)	95% CI (%)	*p*-Value	Mean (%)	95% CI (%)	*p*-Value
Text-Only	Cornea	43.6	[29.1, 59.3]	0.245	74.4	[58.6, 85.6]	0.0596
Figures		30.8	[18.6, 46.5]		53.8	[38.5, 68.4]	
Text-Only	General Medicine	50.0	[5.9, 94.1]	1.00	100	[100, 100]	0.317
Figures		50.0	[9.5, 90.5]		50.0	[9.5, 90.5]	
Text-Only	Glaucoma	66.7	[37.6, 86.9]	0.358	66.7	[37.6, 86.9]	1.00
Figures		83.3	[46.8, 91.1]		66.7	[39.1, 86.2]	
Text-Only	Lens and Cataract	44.4	[17.7, 74.9]	0.644	66.7	[33.3, 88.9]	0.355
Figures		55.6	[26.7, 81.1]		44.4	[12.1, 64.6]	
Text-Only	Neuro-ophthalmology	38.8	[28.0, 50.9]	0.280	58.2	[46.2, 69.4]	0.724
Figures		29.9	[20.2, 41.7]		61.2	[49.2, 72.0]	
Text-Only	Oculoplastics	38.7	[23.5, 56.5]	0.602	74.2	[56.3, 86.5]	0.576
Figures		32.3	[18.6, 49.9]		67.7	[47.0, 78.9]	
Text-Only	Pathology and Tumors	72.1	[57.0, 83.4]	**0.0049**	67.4	[52.3, 79.7]	0.812
Figures		41.9	[28.4, 56.7]		69.8	[54.9, 81.4]	
Text-Only	Pediatrics	53.6	[40.6, 66.1]	**0.013**	62.5	[49.2, 74.1]	0.698
Figures		30.4	[19.9, 43.3]		58.9	[44.1, 69.2]	
Text-Only	Retina and Vitreous	49.3	[39.4, 59.2]	0.360	60.7	[50.6, 69.9]	0.433
Figures		42.7	[32.3, 51.7]		55.1	[44.2, 63.8]	
Text-Only	Uveitis	38.8	[28.0, 50.9]	0.724	55.2	[43.2, 66.6]	0.729
Figures		41.8	[30.7, 53.7]		52.2	[39.1, 62.3]	

Bolded values were statistically significant.

**Table 2:** Comparing the performance of GPT-4 using figures without figure descriptions and GPT-4 using text-only data on diagnosis and next-step tasks by subspecialty.

Prompting Data	Subspeciality	Diagnosis			Next Step		
		Mean (%)	95% CI (%)	*p*-Value	Mean (%)	95% CI (%)	*p*-Value
Text-Only	Cornea	43.6	[29.1, 59.3]	0.258	74.4	[58.6, 85.6]	0.326
Figures + Descriptions		56.4	[38.6, 68.4]		64.1	[45.9, 75.1]	
Text-Only	General Medicine	50.0	[5.9, 94.1]	0.317	100	[100, 100]	1.00
Figures + Descriptions		100	[34.2, 100.0]		100	[34.2, 100.0]	
Text-Only	Glaucoma	66.7	[37.6, 86.9]	0.653	66.7	[37.6, 86.9]	0.219
Figures + Descriptions		75.0	[46.8, 91.1]		41.7	[19.3, 68.0]	
Text-Only	Lens and Cataract	44.4	[17.7, 74.9]	0.637	66.7	[33.3, 88.9]	0.343
Figures + Descriptions		55.6	[26.7, 81.1]		44.4	[12.1, 64.6]	
Text-Only	Neuro-ophthalmology	38.8	[28.0, 50.9]	0.223	58.2	[46.2, 69.4]	0.596
Figures + Descriptions		49.3	[37.7, 60.9]		62.7	[50.7, 73.3]	
Text-Only	Oculoplastics	38.7	[23.5, 56.5]	0.569	74.2	[56.3, 86.5]	0.111
Figures + Descriptions		32.3	[18.6, 49.9]		54.8	[34.8, 68.0]	
Text-Only	Pathology and Tumors	72.1	[57.0, 83.4]	**0.0270**	67.4	[52.3, 79.7]	0.651
Figures + Descriptions		48.8	[32.5, 61.1]		62.8	[47.9, 75.6]	
Text-Only	Pediatrics	53.6	[40.6, 66.1]	0.257	62.5	[49.2, 74.1]	0.552
Figures + Descriptions		42.9	[30.8, 55.9]		67.9	[54.8, 78.6]	
Text-Only	Retina and Vitreous	49.3	[39.4, 59.2]	0.665	60.7	[50.6, 69.9]	0.465
Figures + Descriptions		52.1	[42.2, 61.8]		55.2	[44.2, 63.8]	
Text-Only	Uveitis	38.8	[28.0, 50.9]	0.296	55.2	[43.2, 66.6]	1.00
Figures + Descriptions		47.8	[36.3, 59.5]		55.2	[41.9, 65.1]	

Bolded values were statistically significant.

## Data Availability

The original contributions presented in this study are included in the article/supplementary material. Further inquiries can be directed to the corresponding author.

## Supplementary Materials

The following supporting information can be downloaded at: https://www.mdpi.com/article/10.3390/jpm15040160/s1, Figure S1: Comparison of the number and proportion of cases across different subspecialties that remained correct, became correct, or became incorrect following the inclusion of figures; Figure S2: Comparison of the number and proportion of cases that remained correct, became correct, or became incorrect based on the number of images per figure when figures were included; Figure S3: Comparison of the number and proportion of cases that remained correct, became correct, or became incorrect based on the imaging modality of figures with one image that were included; Figure S4: Comparison of the number and proportion of cases that remained correct, became correct, or became incorrect based on the imaging modality of figures with two images that were included; Figure S5: Comparison of the number and proportion of cases that remained correct, became correct, or became incorrect based on the imaging modality of figures with two images that were included; Table S1: Comparison of the quantity and accuracy of follow-ups by GPT-4 using figures with and without descriptions; Table S2: Comparing the performance of GPT-4 using figures with and without descriptions on diagnosis and next-step tasks by subspeciality; Table S3: Comparing GPT-4 using figures without descriptions with GPT-4 using text-only data on the diagnosis and next-step tasks; Table S4: Comparing GPT-4 using figures and their descriptions with GPT-4 using text-only data on the diagnosis and next0step tasks; Table S5: Comparing GPT-4 using figures with and without descriptions on the diagnosis and next-step tasks.
