# Supplementary material for: Multimodal Performance of GPT-4 in Complex Ophthalmology Cases
Source: J Pers Med. 2025 Apr 21;15(4):160. doi: 10.3390/jpm15040160 (PMC12028970; doi:10.3390/jpm15040160)
Supplement: Supplementary file 1 [file jpm-15-00160-s001.zip › jpm-3433033-supplementary.pdf]

Supplemental figures

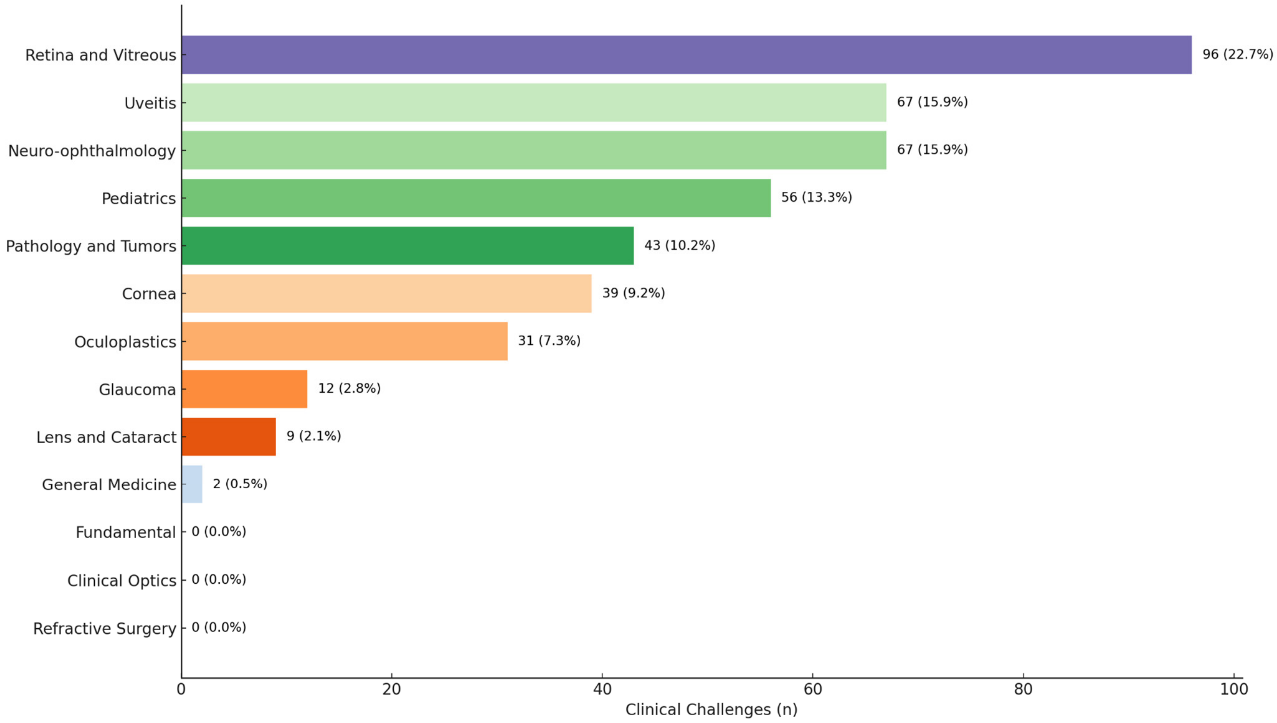

Supplemental Figure S1. JAMA ophthalmology clinical challenge cases by subspeciality.

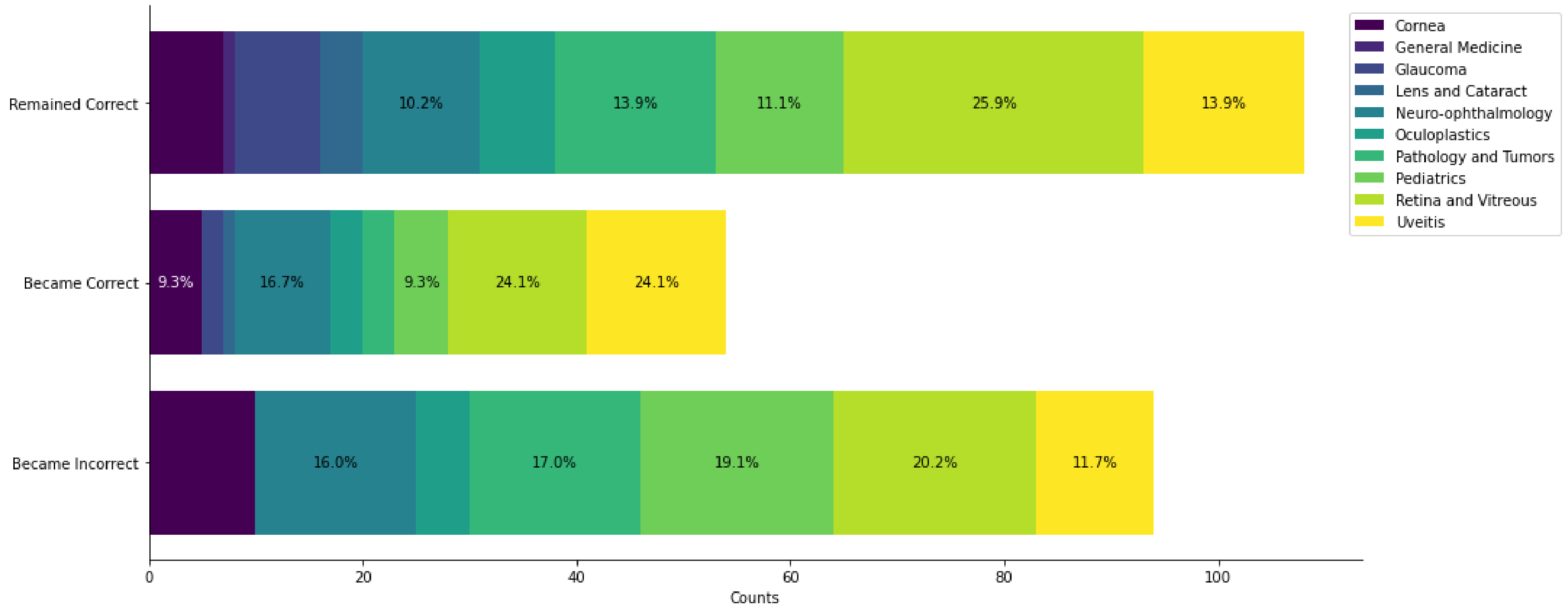

Supplemental Figure S2. Comparison of the number and proportion of cases across different subspecialties that remained correct, became correct, or became incorrect following the inclusion of figures.

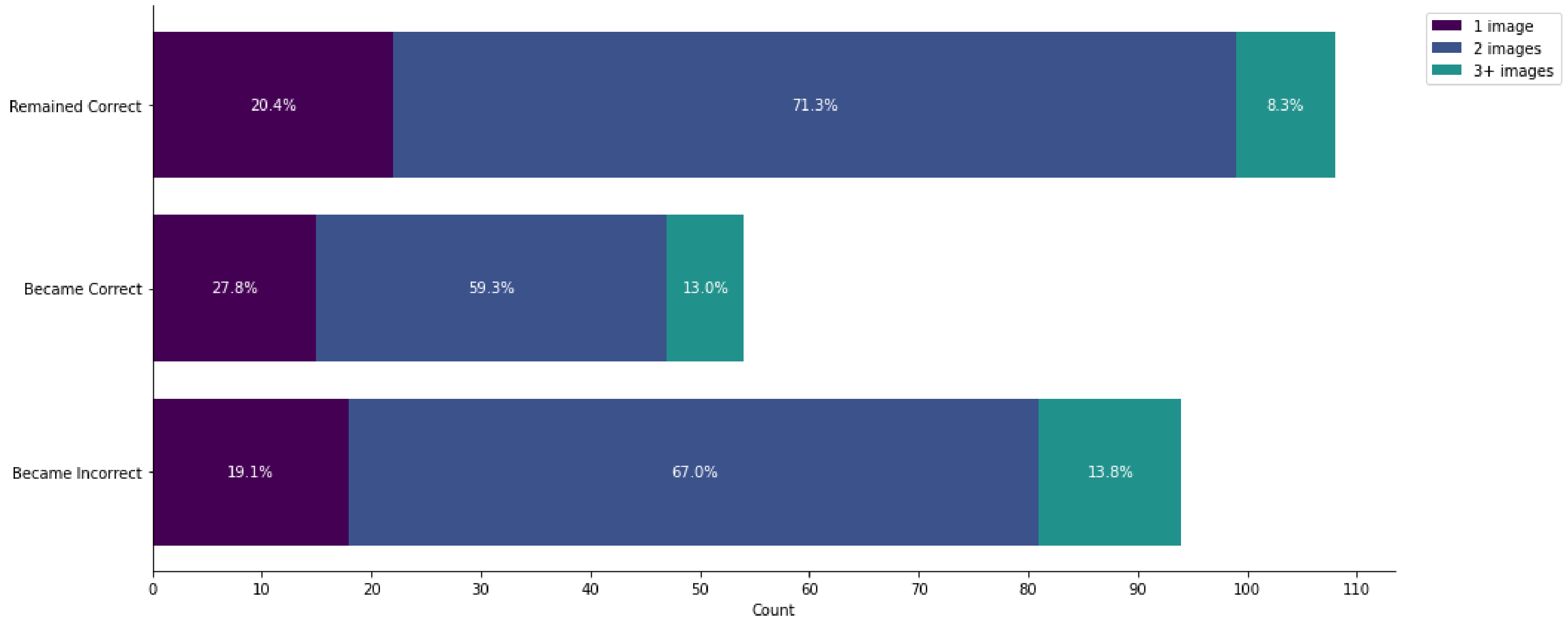

Supplemental Figure S3. Comparison of the number and proportion of cases that remained correct, became correct, or became incorrect based on the number of images per figure when figures were included.

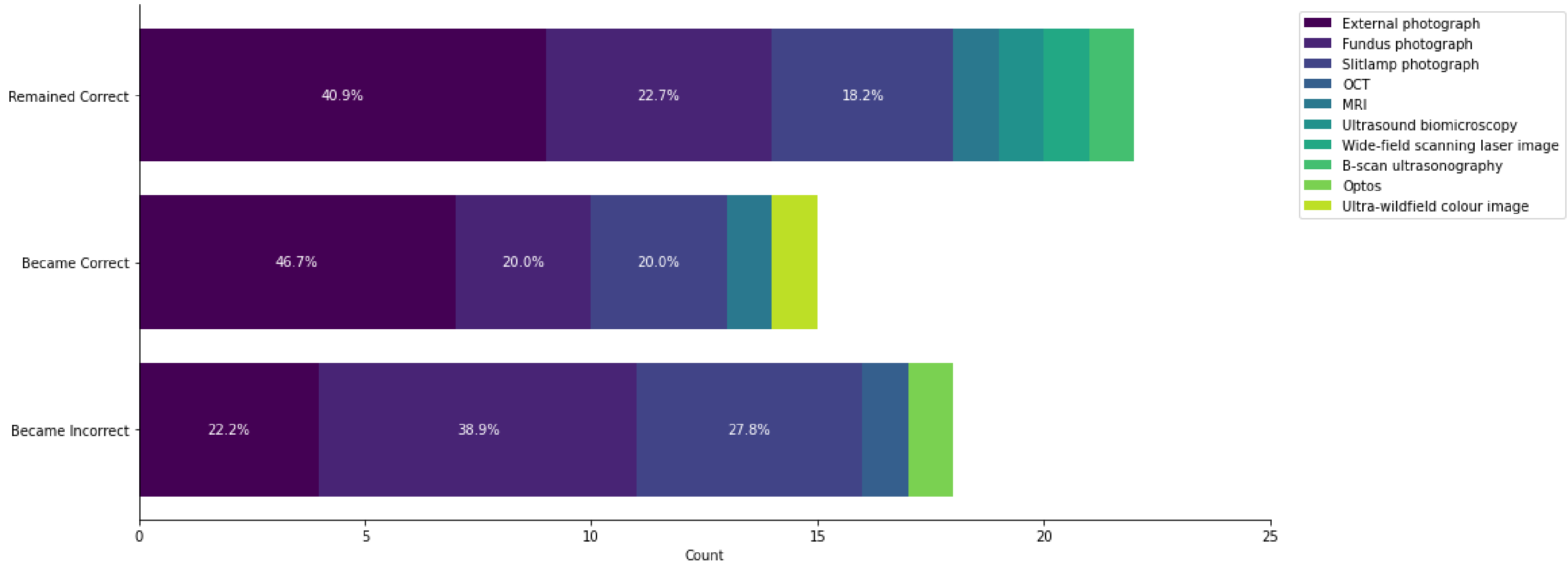

Supplemental Figure S4. Comparison of the number and proportion of cases that remained correct, became correct, or became incorrect based on the imaging modality of figures with one image that were included.

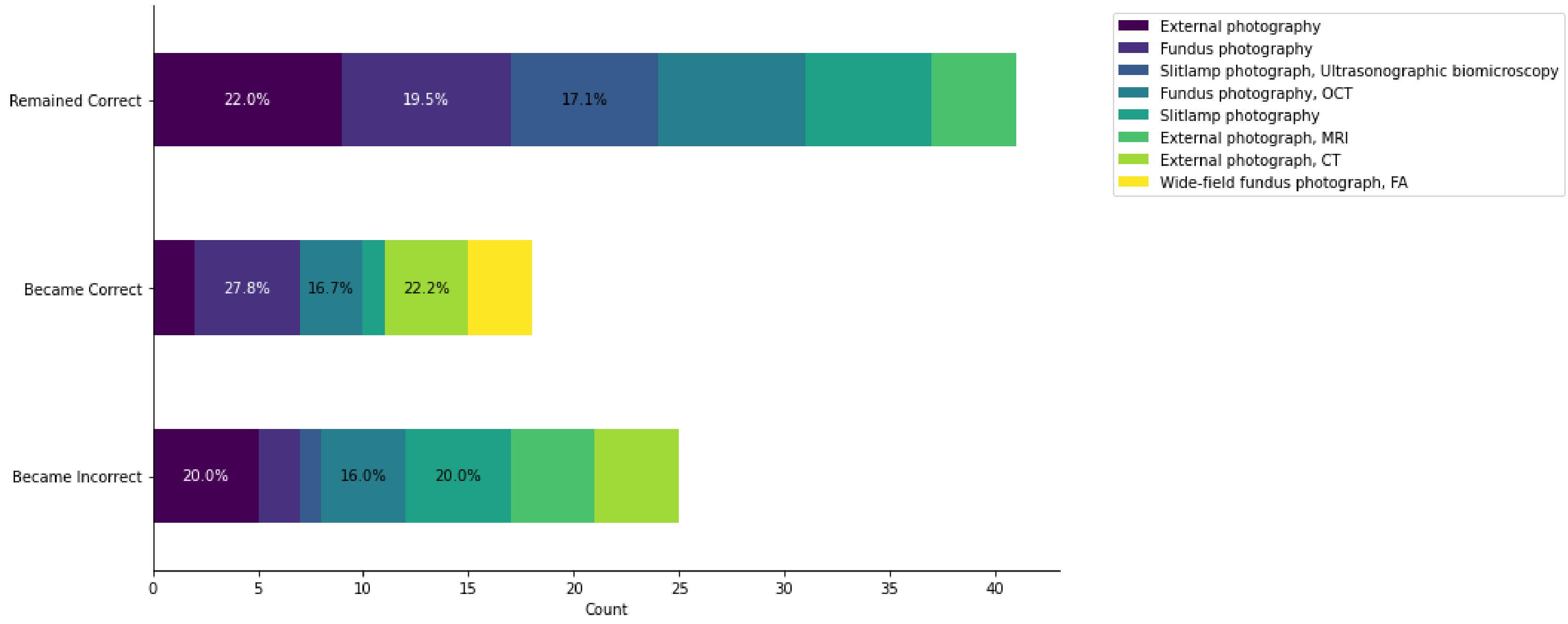

Supplemental Figure S5. Comparison of the number and proportion of cases that remained correct, became correct, or became incorrect based on the imaging modality of figures with two images that were included.

Supplemental tables

Supplemental Table S1. Comparison of the quantity and accuracy of follow-ups by GPT-4 using figures with and without descriptions.

Subspeciality	Figure Descriptions?	Follow-up Prompts Required		Correctly Diagnosed Cases after Follow-up	
		Count (%)	p-value	Count (%)	p-value
Cornea	Yes	17 (43.6)	0.818	9 (52.9)	0.410
	No	15 (38.5)		5 (33.3)	
General Medicine	Yes	1 (50.0)	1.00	1 (100.0)	NA
	No	1 (50.0)		0 (0.00)	
Glaucoma	Yes	2 (16.7)	1.00	1 (50.0)	1.00
	No	2 (16.7)		1 (50.0)	
Lens and Cataract	Yes	3 (33.3)	0.453	0 (0.00)	NA
	No	1 (11.1)		0 (0.00)	
Neuro-ophthalmology	Yes	32 (47.8)	0.160	13 (40.6)	0.159
	No	24 (35.8)		5 (20.8)	
Oculoplastics	Yes	19 (61.3)	0.608	5 (26.3)	0.765
	No	17 (54.8)		3 (17.6)	
Pathology and Tumors	Yes	28 (65.1)	0.661	14 (50.0)	0.323
	No	25 (58.1)		10 (40.0)	
Pediatrics	Yes	32 (57.1)	**0.037**	10 (31.3)	0.977
	No	20 (35.7)		3 (15.0)	
Retina and Vitreous	Yes	45 (46.9)	0.188	18 (40.0)	0.849
	No	35 (36.5)		16 (45.7)	
Uveitis	Yes	39 (58.2)	0.489	18 (46.2)	1.00
	No	34 (50.7)		14 (41.2)	
Total	Yes	218 (51.7)	**0.002**	89 (40.8)	0.149
	No	174 (41.2)		57 (32.8)	

NA Not Applicable

Supplemental Table S2. Comparing the performance of GPT-4 using figures with and without descriptions on diagnosis and next-step tasks by subspeciality.

Subspeciality	Diagnosis p-value	Figure descriptions in higher performer?	Next Step p-value	Figure descriptions in higher performer?
Cornea	**0.0235**	Yes	0.358	Yes
General Medicine	0.317	Yes	0.317	Yes
Glaucoma	0.624	No	0.229	No
Lens and Cataract	1.00	NA	1.00	NA
Neuro-ophthalmology	**0.0222**	Yes	0.859	Yes
Oculoplastics	1.00	NA	0.301	No
Pathology and Tumors	0.523	Yes	0.495	No
Pediatrics	0.172	Yes	0.325	Yes
Retina and Vitreous	0.193	Yes	0.989	Yes
Uveitis	0.487	Yes	0.729	NA

NA Not Applicable

Supplemental Table S3. Comparing GPT-4 using figures without descriptions with GPT-4 using text-only data on the diagnosis and next step tasks.

Diagnosis Task	***p*=0.007**		**GPT-4 (Figures without Descriptions)**		
			**Correct**	**Incorrect**	**Total**
	**GPT-4 (Text-Only)**	**Correct**	108 (25.6%)	94 (22.3%)	202 (47.9%)
		**Incorrect**	54 (12.8%)	166 (39.3%)	220 (52.1%)
		**Total**	162 (38.4%)	260 (61.6%)	422 (100.0%)
Next Step Task	***p*=0.140**		**GPT-4 (Figures without Descriptions)**		
			**Correct**	**Incorrect**	**Total**
	**GPT-4 (Text-Only)**	**Correct**	198 (46.9%)	68 (16.1%)	266 (63.0%)
		**Incorrect**	46 (10.9%)	110 (26.1%)	156 (37.0%)
		**Total**	244 (57.8%)	178 (42.2%)	422 (100.0%)

GPT-4 Generative Pre-trained Transformer 4

Supplemental Table S4. Comparing GPT-4 using figures and their descriptions with GPT-4 using text-only data on the diagnosis and next step tasks.

Diagnosis Task	***p*=0.684**		**GPT-4 (Figures with Descriptions)**		
			**Correct**	**Incorrect**	**Total**
	**GPT-4 (Text-Only)**	**Correct**	145 (34.4%)	57 (13.5%)	202 (47.9%)
		**Incorrect**	63 (14.9%)	157 (37.2%)	220 (52.1%)
		**Total**	208 (49.3%)	214 (50.7%)	422 (100.0%)
Next Step Task	***p*=0.258**		**GPT-4 (Figures with Descriptions)**		
			**Correct**	**Incorrect**	**Total**
	**GPT-4 (Text-Only)**	**Correct**	210 (49.8%)	56 (13.3%)	256 (63.0%)
		**Incorrect**	40 (9.5%)	116 (27.5%)	156 (37.0%)
		**Total**	250 (59.2%)	172 (40.8%)	422 (100.0%)

GPT-4 Generative Pre-trained Transformer 4

Supplemental Table S5. Comparing GPT-4 using figures with and without descriptions on the diagnosis and next step tasks.

			GPT-4 (Figures without Descriptions)		
	***p*=0.0014**		**Correct**	**Incorrect**	**Total**
Diagnosis Task	**GPT-4 (Figures with Descriptions)**	**Correct**	117 (27.7%)	91 (21.6%)	208 (49.3%)
		**Incorrect**	45 (10.7%)	169 (40.0%)	214 (50.7%)
		**Total**	162 (38.4%)	260 (61.6%)	422 (100.0%)
			**GPT-4 (Figures without Descriptions)**		
	***p*=0.680**		**Correct**	**Incorrect**	**Total**
Next Step Task	**GPT-4 (Figures with Descriptions)**	**Correct**	196 (46.4%)	54 (12.8%)	250 (59.2%)
		**Incorrect**	48 (11.4%)	124 (29.4%)	172 (40.8%)
		**Total**	244 (57.8%)	178 (42.2%)	422 (100.0%)

GPT-4 Generative Pre-trained Transformer 4
